# Accuracy of ultrasonography for differentiating between simple and complex appendicitis in children

**DOI:** 10.1007/s00383-021-04872-8

**Published:** 2021-03-07

**Authors:** David J. Nijssen, Paul van Amstel, Joost van Schuppen, Laurens D. Eeftinck Schattenkerk, Ramon R. Gorter, Roel Bakx

**Affiliations:** 1 Department of Pediatric Surgery, Amsterdam UMC, Emma Children’s Hospital, University of Amsterdam and Vrije Universiteit Amsterdam, P.O. Box 22660, 1100 DD Amsterdam, The Netherlands; 2 Department of Radiology and Nuclear Medicine Amsterdam UMC, University of Amsterdam, Amsterdam, The Netherlands

**Keywords:** Appendicitis, Complex appendicitis, Ultrasonography, Pediatric surgery, Perforated appendicitis, Appendectomy

## Abstract

**Purpose:**

Accurate differentiation between simple and complex appendicitis is important since differences in treatment exist. This study aimed to assess the accuracy of ultrasonography in differentiating between simple and complex appendicitis.

**Methods:**

Data from children aged < 18 years who underwent appendectomy between the 1st of January 2013 and the 1st of January 2018 were analyzed retrospectively. Ultrasonography reports of eligible children were divided into simple (test negative) and complex appendicitis (test positive) based on predefined criteria and compared to a gold standard (a combination of predefined perioperative and histopathological criteria). Sensitivity, specificity, negative predictive value (NPV) and positive predictive value (PPV) were calculated to measure ultrasonographic accuracy in differentiating between simple and complex appendicitis.

**Results:**

176 children were eligible for inclusion. The mean age at the time of operation was 10.1 ± SD 4.6 years. 84 (47.7%) children had simple appendicitis and 92 (52.3%) had complex appendicitis. The use of ultrasonography yielded a sensitivity: 46%, specificity: 90%, PPV: 84%, and NPV: 60%.

**Conclusion:**

Ultrasonography as standalone modality is not suitable for differentiating between simple and complex appendicitis in children. To improve preoperative differentiation, other variables such as clinical signs and laboratory data are necessary in conjunction with ultrasonography findings.

## Introduction

Changing insights into the pathogenesis of acute appendicitis have led to the distinction of two types of appendicitis, simple and complex. The idea that acute appendicitis is a progressive disease, irreversibly leading to perforation with generalized peritonitis has been debated over the past years. The awareness of a type of appendicitis without the tendency of perforation led to the discussion of whether or not an appendectomy is mandatory for all patients with acute appendicitis.

In the last few years, more and more studies reported their outcome of a non-operative treatment strategy for patients with simple appendicitis. Overall, these studies show that a non-operative treatment strategy is safe and able to avoid an appendectomy in ± 75% of the children at 1-year follow-up [1–3]. Additional benefits are a potential decrease in complications, utilization of pain medication and reduction in costs [3, 4]. Furthermore, studies have shown that primary failure of the non-operative treatment strategy is partly due to misdiagnosis of complex appendicitis [1, 5]. Therefore, selection of eligible patients (i.e. patients with simple appendicitis) for a non-operative treatment strategy is crucial to further increase its effectiveness. In the diagnostic work-up of children with suspected acute appendicitis, attention should be given to the assumed type of appendicitis (i.e. simple or complex) to improve the selection of eligible patients for non-operative treatment.

Ultrasonography plays a central role in the diagnostic work-up of children with suspected appendicitis. The implementation of ultrasonography as standard modality in the diagnostic work-up has resulted in a significant reduction of negative appendectomies [6, 7]. However, data regarding its potential in discriminating between simple and complex appendicitis in the pediatric population are scarce. Therefore, the aim of this study is to evaluate the diagnostic accuracy of ultrasonography in differentiating between simple and complex acute appendicitis in a cohort of children treated at our academic hospital.

## Methods

### Patients

In this single-center retrospective diagnostic test study, we included children aged 0–17 years with an ultrasonographic confirmed diagnosis of acute appendicitis that underwent appendectomy at our pediatric surgical center (tertiary referral center) between the 1st of January 2013 and the 31st of December 2017. Patients were identified using specific care activity codes for laparoscopic appendectomy (034911) and open appendectomy (034910). Patients who were diagnosed with appendicitis in another hospital and were transferred for appendectomy to our center and those that underwent appendectomy elsewhere were excluded. Patients who underwent appendectomy for another indication than acute appendicitis, patients with a non-inflamed appendix (histologically proven in retrospect), and those with missing data regarding ultrasonography, surgery or histopathology reports were also excluded.

Our Research Ethics Committee declared that the Medical Research involving Human Subjects Act (WMO) did not apply to this study and, therefore, no official approval was required by national law.

### Data extraction and definitions

For all eligible patients, data regarding baseline characteristics (i.e. age, gender), ultrasonography, surgery, and histopathology were extracted from the electronic patient files according to a standardized form. Data extraction and classification of the type of appendicitis was performed by one author (DJN) and another author (RB) randomly checked 20% of all patients. In case of disagreement regarding the type of appendicitis (simple or complex), a third expert (RRG) was consulted. Ultrasonography reports were classified (in retrospect) as either indicative of simple or complex appendicitis. Ultrasonographic criteria for complex appendicitis were: peri-appendiceal purulent-free fluid of more than 1 cm in diameter, extraluminal gas/air, paralytic ileus, (local or diffuse) loss of the submucosal layer of the appendix, and signs of appendiceal abscess/phlegmon. In case one of these criteria was found on ultrasonography, the report was classified as indicative of complex appendicitis. Ultrasonography images without signs of complex appendicitis were classified as indicative of simple appendicitis (see Table 1). Classification was based on the written reports, the images were not reassessed.

**Table 1:** Sonographic criteria for simple and complex appendicitis

Sonographic simple appendicitis	Sonographic complex appendicitis
Incompressible appendix Outer diameter of ≥ 6 mm Hyperemic appendiceal wall Infiltration of peri-appendiceal fat No signs of perforation or abscess/phlegmon	Purulent free fluid > 1 cm diameter Extraluminal gas/air Paralytic ileus Loss of submucosal layer Sings of abscess/phlegmon

Based upon perioperative and histopathological findings, patients were ultimately divided into two groups according to the classification by Bhangu [8]:

- Simple appendicitis: intraoperative signs of congestion, an increased diameter, (red) color change, exudate or pus; or histopathologic signs of transmural inflammation, ulceration, or thrombosis, with or without extramural pus.
- Complex appendicitis: perioperative signs of a friable appendix with purple, green or black color changes, a visible perforation, and/or abscess formation, or histopathologic signs of transmural inflammation with signs of necrosis or perforation.

Results of ultrasonography reports (classified as either indicative of simple or complex appendicitis) were compared to the perioperative/histopathological classification, which was used as our gold standard test.

### Outcomes

The primary outcome was the diagnostic accuracy of ultrasonography in differentiating between simple and complex appendicitis. Diagnostic accuracy was assessed by obtaining sensitivity and specificity, as well as the positive predictive value (PPV) and negative predictive value (NPV). We stratified by gender to investigate whether these groups differed in our primary outcome.

The secondary outcome was the interrater variability between (pediatric) radiologists (or residents under the supervision of a pediatric radiologist) and radiology residents (without supervision) with regard to the ultrasonographic diagnosis of complex appendicitis.

### Used materials

Abdominal ultrasonography was performed on a Philips UI22 (Philips Medical Systems, Best, The Netherlands), with a 5 or 9 MHz curved probe or 12 MHz linear probe, with appropriate pediatric abdominal settings.

### Data analysis

Descriptive statistics were performed using IBM SPSS version 23.0 (IBM Corp., Armonk, NY, USA). A comparison of ultrasonography to our gold standard is displayed in a 2 × 2 contingency table. Complex appendicitis was considered as a positive test result in our index test (ultrasonography) and our gold standard test. Accordingly, simple appendicitis was considered as a negative test result in both tests. Using the 2 × 2 contingency table, our primary outcome measure was calculated.

Secondary, a Chi-squared test was performed to assess the interrater variability of radiologists and radiology residents. Statistical significance was defined as a *p* value < 0.05.

## Results

**Fig. 1:**
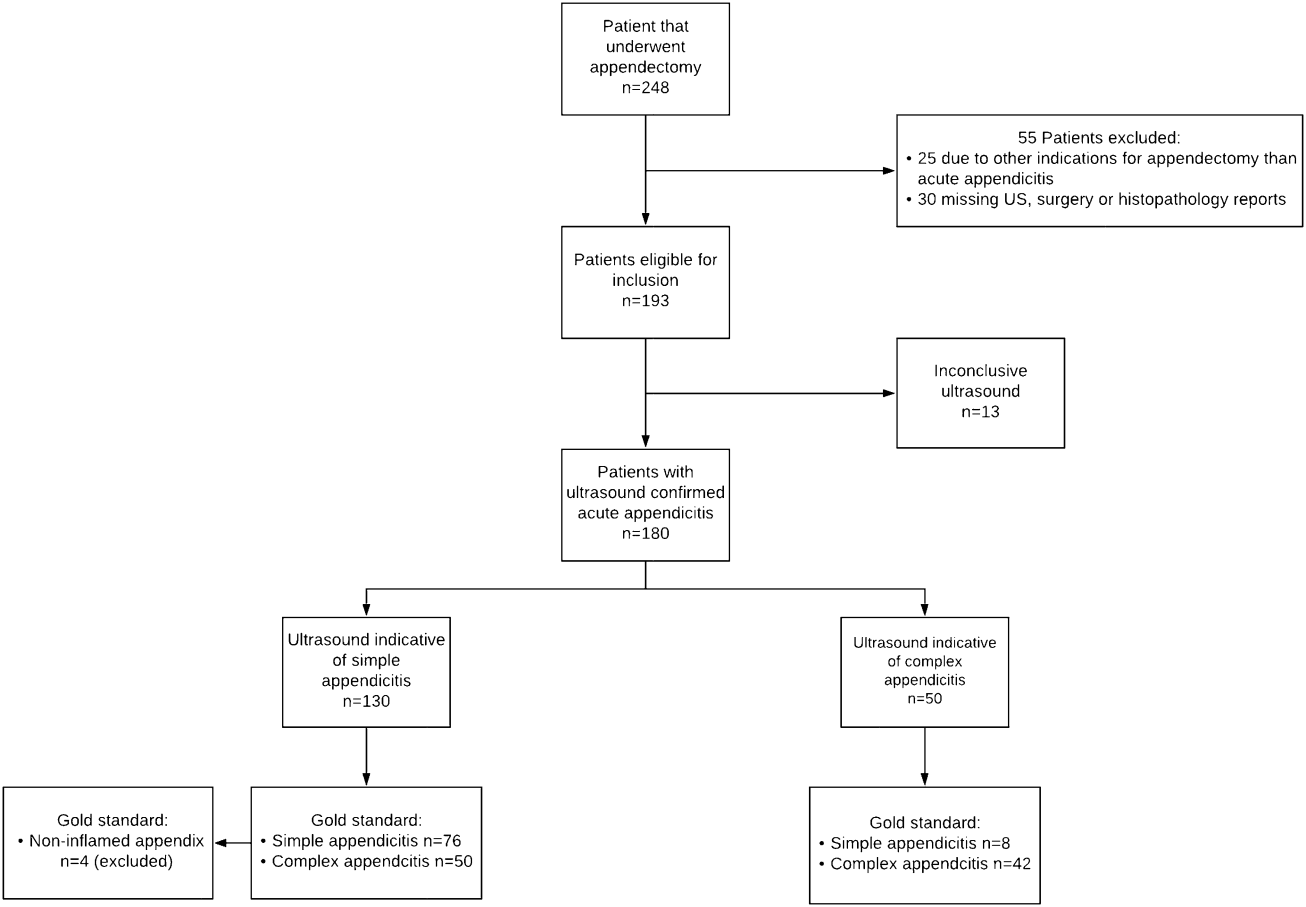
Patient selection

During the study period, 248 patients underwent appendectomy at our pediatric surgical center. Of these, 72 patients were excluded from analysis due to various reasons. See Fig. 1 for a flowchart of exclusions with reasons. A total of 176 patients were included with a mean age (SD) at time of operation of 10.10 (± 4.6) years. Of these, 98 patients were male (56%) and 78 were female (44%).

### Accuracy of ultrasonography

Ultrasonography reports of 50 (28.4%) patients were classified as indicative of complex appendicitis (test positive) and 126 (71.6%) patients as indicative of simple appendicitis (test negative). The most frequent reason for a complex ultrasonography result was the presence of peri-appendiceal free fluid > 1 cm diameter, suggestive of a perforation (without abscess formation) in 27 (54%) cases, followed by perforation with abscess formation in 14 (28%) and solely abscess formation in 9 (18%).

According to our gold standard 92 (52.3%) patients were diagnosed with complex appendicitis and 84 (47.7%) with simple appendicitis. Table 2 displays the 2 × 2 contingency table derived from these results. Accordingly, a sensitivity of 46% (95% CI 35–56%) and a specificity of 90% (95% CI 82–96%) was found for the accuracy of ultrasonography in differentiating complex from simple appendicitis. 84% (95% CI 72–91%) of patients with an ultrasonography report indicative of complex appendicitis were diagnosed with complex appendicitis according to our gold standard (PPV). Of those patients with an ultrasonography report indicative of simple appendicitis, 60% (95% CI 55–65%) were diagnosed with simple appendicitis according to our gold standard (NPV). After stratification by gender (Tables 3 and 4), we calculated the same diagnostic test accuracy for males [sensitivity: 44% (95% CI 30–59%), specificity: 96% (95% CI 86–100%), PPV: 91% (95% CI 72–97%), NPV: 64% (95% CI 58–70%)] and females [sensitivity: 48% (95% CI 32–63%), specificity: 82% (95% CI 65–93%), PPV: 78% (95% CI 61–89%), NPV: 55% (95% CI 47–63%)].

**Table 2:** Comparison ultrasonography (US) with gold standard

	Gold standard result		Total
	Complex	Simple	
US result			
Complex	42	8	50
Simple	50	76	126
Total	92	84	176

**Table 3:** Comparison ultrasonography (US) with gold standard in males

	Gold standard result		Total
	Complex	Simple	
US result			
Complex	21	2	23
Simple	27	48	75
Total	48	50	98

**Table 4:** Comparison ultrasonography (US) with gold standard in females

	Gold standard result		Total
	Complex	Simple	
US result			
Complex	21	6	27
Simple	23	28	51
Total	44	34	78

### Interrater variability

Table 5 shows the interrater variability between (pediatric) radiologists and radiology residents with regard to the diagnosis of complex appendicitis. For 81 of the total of 92 patients with complex appendicitis according to our gold standard, data on who performed ultrasonography were available. Using this data, a comparison was made between the performance of the two different observer groups: (pediatric) radiologists (or residents under the supervision of a pediatric radiologist) and radiology residents. This showed that there was no significant difference in diagnostic accuracy between the observers (*p* = 0.911).

**Table 5:** Comparison ultrasonography (US) performance for diagnosing complex appendicitis

	US performer		Total
	Specialist	Resident	
US result			
Complex	26	10	36
Simple	33	12	45
Total complex cases by gold standard	59	22	81

## Discussion

In this study, we aimed to evaluate the accuracy of ultrasonography for differentiating between simple and complex appendicitis in children. The diagnosis of complex appendicitis on ultrasonography was associated with a high probability of finding complex appendicitis by the gold standard test. However, when simple appendicitis was found on ultrasonography, more than half of the patients had a confirmed complex appendicitis by gold standard, showing that ultrasonography is poorly accurate to rule out complex appendicitis. Additionally, subgroup analysis showed that outcomes were comparable for males and females. If decision making would be solely based on ultrasonography, many cases in this cohort would have been misclassified as simple appendicitis. Therefore, this study showed that in our tertiary pediatric center, the standalone use of ultrasonography for the differentiation between simple and complex appendicitis is not suitable.

Studies that assess the overall diagnostic accuracy of ultrasonography for differentiating between simple and complex appendicitis in the pediatric population are scarce. Other studies either investigated a cohort of children with suspected appendicitis, including children with other diagnoses as well, or examined the predictive accuracy of individual ultrasonographic variables to diagnose simple and complex appendicitis [9–11]. Reported diagnostic accuracy of variables such as appendiceal wall diameter and echogenic loss of submucosal layer yielded a sensitivity and specificity of more than 90% [10, 11]. One of these studies even found a perfect sensitivity of 100% using echogenic loss of submucosal layer as a predictive variable for complex appendicitis [10]. Some of these individual ultrasonographic variables show promising results for the detection of complex appendicitis in children and its implementation in routine diagnostic work-up is worth exploring. Unfortunately, we were not able to investigate the value of these specific ultrasonographic criteria, as in our center, these criteria were not routinely described in ultrasonography reports.

Secondary, in our cohort, the ability to detect complex appendicitis on ultrasonography was not significantly different for radiology residents compared to (pediatric) radiologists. Previous studies regarding the influence of the level of experience on the diagnostic accuracy of ultrasonography showed contradicting results. Some studies found a significant interobserver variability for the assessment of ultrasonography images to differentiate simple and complex appendicitis and, therefore, suggested that the level of experience of the observer may be of influence [7, 9]. Other researchers, however, found a moderate (0.4 < Kappa ≤ 0.6) to high (Kappa > 0.6) agreement between observers when assessing ultrasonography for perforated appendicitis [12]. Standardization of ultrasonography performance and assessment may reduce interobserver variability. Furthermore, this would create an opportunity to incorporate the aforementioned ultrasonographic variables with a high potential to detect complex appendicitis into a routine ultrasonography procedure.

Recent awareness of a type of appendicitis without the tendency of perforation and the subsequent interest in non-operative treatment of this type of appendicitis has increased the importance of an accurate preoperative test for the selection of patients that are eligible for a non-operative treatment strategy. Especially since non-operative treatment failure (occurring in approximately 10% of patients) is mostly due to misdiagnosis of simple appendicitis [1, 5]. This indicates the importance of an accurate test that is capable of differentiating between simple and complex appendicitis. However, additional factors such as the presence of an appendicolith, influence the outcome of non-operative treatment. The presence of an appendicolith is associated with both a higher primary failure rate of non-operative treatment and a higher recurrence rate of acute appendicitis after non-operative treatment [13]. These results show that, apart from the differentiation between simple and complex appendicitis, other radiological variables have to be taken into consideration when selecting patients for a non-operative treatment strategy.

Management protocols of many countries rely heavily on CT-scans as primary modality for the detection of acute appendicitis in children [14]. Although the CT-scan has proven to be capable of detecting appendicitis, several studies have shown that a CT-scan lacks the capability of accurate differentiation between simple and complex appendicitis. A recent systematic review showed that the discriminating properties of a CT-scan are similar to ultrasonography (i.e. highly specific but non-sensitive) [15]. This lack in discriminative ability was also reported by a randomized controlled trial comparing surgery and conservative treatment for patients with a CT-scan confirmed diagnosis of simple appendicitis. This study found that in 18% of patients that were classified as simple appendicitis by CT-scan, signs of complex appendicitis were found perioperative [16]. Major downsides of CT-scans compared to ultrasonography are significant radiation exposure and higher costs. Therefore, our national guideline recommends the use of ultrasonography as primary modality for the diagnosis of acute appendicitis and in case of persistent inconclusive results the performance of an MRI. This imaging strategy reduces the exposure to ionizing radiation and the attendant risk of radiation-induced malignancies [17]. Since the downsides of a CT-scan are outweighed by neither a more accurate diagnosis of acute appendicitis nor a more accurate differentiation between simple and complex appendicitis, in our opinion ultrasonography should be the imaging modality of choice during diagnostic workup of children with a suspicion of acute appendicitis.

As our results and previous studies show that ultrasonography, as currently performed, is unable to accurately differentiate between simple and complex appendicitis in children, we believe that the implementation of a clinical prediction rule that combines ultrasonography results with clinical and laboratory variables might improve distinctiveness. Thus far, several clinical prediction rules have been developed to aid clinicians in differentiating between simple and complex appendicitis in both the pediatric and adult populations [18–20]. Some of these models demonstrated sensitivity and specificity of more than 90%. Although these scoring systems show promising results, most of them have not yet been externally validated in large cohorts. If they are properly validated, these models could aid the selection of children eligible for non-operative treatment in randomized controlled trials.

## Limitations

There are several limitations to this study. Since this study is based on data that are retrospectively collected from patient files, some degree of reporting bias and missing data are inevitable. Moreover, the study could be biased by some degree of interpretative bias which is dependent on the level of experience of the author that extracted the data. Therefore, 20% of the data was checked by a second author and in all cases of doubt, a third highly experienced expert was consulted.

Seventeen patients were excluded from our analysis due to inconclusive ultrasonography or a negative appendectomy, which could have influenced the diagnostic accuracy of ultrasonography in our study. If the aim was to assess the accuracy of ultrasonography in detecting (simple or complex) appendicitis, these patients should have been included in the analysis. However, this study aimed to investigate the accuracy of ultrasonography in differentiating simple and complex appendicitis in children with ultrasonography confirmed appendicitis. Therefore, it would be methodologically incorrect to include inconclusive ultrasonography reports in our analysis. Including patients with negative appendectomies in our analysis would also be incorrect, as the ultrasonography results of these patients could not be compared to our gold standard, which consisted of a perioperative and histopathological diagnosis of acute appendicitis (classified as simple or complex based on predefined criteria).

Furthermore, ultrasonographic differentiation between simple and complex appendicitis might be hampered by incomplete use of a standardized reporting template, although our radiologists have implemented a standardized ultrasonography report in the last few years. Ultrasonography images were not reassessed by radiologists and thus classification of either simple or complex appendicitis on ultrasonography was based on written reports. However, due to the fact that ultrasonography is a dynamic examination, reassessment of the images would still not guarantee a more accurate discriminating property of ultrasonography. Furthermore, the ultrasounds were performed by a variety of pediatric radiologists and radiology residents. Therefore, this study was influenced by intra- and inter-observer variability, as the individual interpretation of the examination is according to the level of experience of the investigator. Additionally, the generalizability of our results is reduced by the fact that this study included children with acute appendicitis in a tertiary pediatric center, that is focused on treating children with complex gastrointestinal disease. This also explains why we could only include a relatively small cohort of children in this study.

## Conclusion

Ultrasonography as a standalone modality is not suitable to predict whether a child suffers from a simple or complex appendicitis. To improve the classification between the two entities other variables such as clinical signs and laboratory data are necessary in conjunction with ultrasonography findings.
